# Long-term survival and polyclonal immunoglobulin reconstitution after allogeneic stem cell transplantation in multiple myeloma

**DOI:** 10.1007/s00277-020-04068-5

**Published:** 2020-05-22

**Authors:** Christine Eisfeld, Eva Eßeling, Ramona Wullenkord, Cyrus Khandanpour, Julia Reusch, Jan-Henrik Mikesch, Christian Reicherts, Andrea Kerkhoff, Christoph Schliemann, Torsten Kessler, Rolf M. Mesters, Wolfgang E. Berdel, Georg Lenz, Matthias Stelljes

**Affiliations:** Department of Medicine A, University Hospital Münster, Münster, Germany

**Keywords:** Allogeneic hematopoietic stem cell transplantation, Multiple myeloma, Immune reconstitution, Immunoparesis

## Abstract

Despite significant progress made in the treatment of patients with multiple myeloma (MM) in the last decade, for patients with early relapse or rapidly progressing high-risk disease, allogeneic hematopoietic stem cell transplantation (SCT) might be an option leading to long-term survival. Here, we retrospectively analyzed the outcomes of 90 MM patients who received allogeneic SCT in our center between 1999 and 2017. We specifically assessed the association of impaired humoral immune reconstitution, referred to as immunoparesis, and post-transplant survival. Sixty-four patients received allogeneic SCT in relapse following 2–7 lines of therapy; 26 patients received upfront tandem autologous-allogeneic SCT. With a median follow-up of 76 months, OS and PFS were 52.6% (95% CI 42.9–64.3) and 36.4% (95% CI 27.6–47.9) at 2 years and 38.6% (95% CI 29.2–51.1) and 25.3% (95% CI 17.5–36.4) at 5 years, respectively. Receiving more than two therapy lines prior to transplantation was an independent risk factor for OS (HR 3.68, 95% CI 2.02–6.70) and PFS (HR 3.69, 95% CI 2.09–6.50). In a landmark analysis at day 200, prolonged immunoparesis was associated with reduced OS (HR 3.22, 95% CI 1.14–9.11). Allogeneic stem cell transplantation offers an additional treatment element that may lead to long-term remission in selected patients with poor prognosis, probably exploiting graft-versus-myeloma effects. Immunoparesis could potentially serve as an indicator for impaired survival following allogeneic transplantation, an observation to be further studied prospectively.

## Introduction

Although the range of therapeutic options for patients with multiple myeloma (MM) increased substantially, for the majority of younger patients, the backbone of triple induction therapy, consolidation with high-dose melphalan, and autologous stem cell transplantation (SCT) followed by maintenance treatment leads to prolonged disease remission and remains the standard of care [1]. In a subset of patients with International Staging System (ISS) stage III disease or high-risk cytogenetic abnormalities, though, remission is often limited to few months, and subsequent treatments including new agents often fail to induce deep and long-lasting remissions [2]. Allogeneic SCT potentially allows long-term survival and has shown to overcome the prognostic impact of high-risk cytogenetics in several nonrandomized studies [3–5]. Despite improvements in transplantation procedures and supportive therapy, though, relapse occurs in 35–72% within the first 2 years after allogeneic SCT, and non-relapse mortality is a main concern [3, 6, 7]. Pre-transplant characteristics with predictive significance for survival have been identified, whereas decisions on post-transplant management are mainly based on retrospective case series. Maintenance therapy including immunomodulatory substances and pre-emptive donor lymphocyte infusion (DLI) may enhance the graft-versus-myeloma effect and improve disease control [8–10]. However, evidence to support clinical guidance on when and in whom to start maintenance or pre-emptive therapy is lacking.

The beneficial role of timely cellular immune reconstitution after allogeneic SCT on outcomes has been outlined elsewhere [11–13]. Besides preventing from fatal infections, strong and early immune reconstitution may enhance the graft-versus-malignancy effect and eliminate residual malignant cells. The assessment of levels of polyclonal immunoglobulins (Ig) potentially provides indirect insights into the B-cellular immune reconstitution. Immunoparesis, defined as suppression of polyclonal Ig uninvolved in the clonal disease, reflects a lower percentage of normal bone marrow plasma cells and is observed in most MM patients at diagnosis, being often reversible under treatment [14, 15]. Persistent immunoparesis in MM patients has shown to be an adverse prognostic factor for patients in remission 1 year after autologous SCT [14, 16]. To our knowledge, kinetics of polyclonal Ig reconstitution has not been systematically evaluated after allogeneic SCT.

## Methods

We retrospectively analyzed patients with MM consecutively undergoing allogeneic peripheral blood stem cell transplantation in our University Medical Center between 1999 and 2017 with respect to patient characteristics, post-transplant strategies, and humoral immune reconstitution.

Patients with plasma cell leukemia were excluded from the analysis. Data were retrieved by electronic records. Written informed consent was available for all patients and the study was approved by the local ethics committee. Remission status was classified according to the International Myeloma Working Group (IMWG) criteria [17]. Overall survival (OS) and progression-free survival (PFS) were defined as time from transplantation to death and progressive disease or death, respectively. Non-relapse mortality (NRM) was defined as death without progressive disease, with relapse considered as a competing risk, and when NRM was given as percentage with the patients treated as the denominator. Cumulative incidence probabilities of NRM and relapse were modeled as described [18].

Mann-Whitney *U* test and *χ*^2^ test were used to compare groups; univariate comparisons were performed with log-rank test for OS and PFS and Gray’s test for cumulative incidence functions. Known risk factors and covariates with significant impact in univariate analysis were included in multivariate Cox proportional hazards regression analysis.

To explore the prognostic role of immunoglobulin reconstitution, levels of the uninvolved immunoglobulins measured by turbidimetry at 100, 200, and 360 days (± 50 days) after transplantation were recorded. Total lymphocyte counts were recorded for the corresponding time points. Immunoparesis was defined as > 25% decrease in one or both uninvolved immunoglobulins relative to the lower limit of normal range [14]. OS and PFS probabilities were evaluated using a landmark approach with the landmarks set at 100, 200, and 360 days after allogeneic SCT, respectively. To exclude direct influence of relapse on immunoglobulin levels, patients with relapse before the respective landmark were excluded. Conditional versions of the Cox model were applied for each landmark. Descriptive statistics were performed using IBM SPSS Statistics version 25.0; survival analysis was performed using the “R” packages “survival” and “cmprsk”.

## Results

### Patient characteristics

A total of 90 patients with MM underwent allogeneic SCT between 1999 and 2017. Patient characteristics are summarized in Table 1. Median age at time of transplantation was 51 years (range 35–68); 64 patients were male. Twenty-six had allogeneic SCT in first-line therapy as part of a tandem autologous-allogeneic transplantation concept, partly within clinical trials (EudraCT Nos. 2007-004928-21 and 2009-016616-21). Sixty-four patients received allogeneic SCT in relapsed or refractory (r/r) disease after at least two lines of therapy.

**Table 1:** Patient and transplant characteristics

Characteristic	*n*
Age at SCT, years	
Median (range)	51 (35–68)
Sex (%)	
Male/female	64 (71)/26 (29)
Myeloma subtype	
IgG	47
IgA	27
IgD	3
FLC only	11
Missing	2
Initial disease stage according to ISS (%)	
I	21 (23)
II	12 (13)
III	15 (17)
Missing	42 (47)
Cytogenetics (%)	
High risk: *t*(4;14), *t*(14;16), *t*(14;20), or del17p	20 (22)
Poor risk: gain 1q or del1p	6 (7)
Time from diagnosis to SCT, months	
Median (range)	22 (7–198)
Number of therapy lines before SCT (%)	
First-line auto-allo	26 (29)
2	25 (28)
3	18 (20)
4	10 (11)
5	5 (6)
6	4 (4)
7	2 (2)
Pre-treatment with PI/IMiD (%)	73 (81)/56 (62)
Donor (%)	
MRD	40 (44)
MUD	39 (43)
Mismatched	11 (12)
Conditioning regimen (%)	
Myeloablative	54 (60)
Reduced intensity	36 (40)
Melphalan-based	34 (38)
Busulfan-based	32 (36)
TBI-based	21 (23)
GvHD prophylaxis (%)	
CSA/MMF/ATG	35 (39)
CSA/MTX/ATG	38 (42)
CSA/MMF	16 (18)
CSA/MTX	1 (1)

*SCT*, allogeneic stem cell transplantation; *ISS*, international staging system; *PI*, proteasome inhibitors; *IMiD*, immunomodulatory drugs; *MRD*, matched related donor; *MUD*, matched unrelated donor; *GvHD*, graft-versus-host disease; *CSA*, cyclosporine A; *MMF*, mycophenolate mofetil; *ATG*, anti-thymocyte globulin; *MTX*, methotrexate

Cytogenetic risk profiling according to the current IMWG consensus criteria [19] has been performed in a minority of patients, as until recently, examination for deletion of 13q14 only has been standard of care. Overall, 26 patients (29%) showed poor or high-risk cytogenetics.

Median time from diagnosis to allogeneic SCT was 22 months (range 7–198) and patients had a median of two (range 1–7) prior lines of therapy. Most patients have been treated with proteasome inhibitors (81%) and/or immunomodulatory drugs (IMiDs) (62%) before transplantation. At the time of allogeneic SCT, 82% of patients had disease control (10% complete remission, 30% very good partial remission, and 41% partial remission).

Forty patients were transplanted from a matched related donor (MRD), 39 patients from a 10/10 matched unrelated donor, and 11 patients from a mismatched donor. All patients received peripheral blood stem cell (PBSC) transplantation. Sixty percent received myeloablative conditioning (MAC) regimens, and in 81%, graft-versus-host disease (GvHD) prophylaxis included anti-thymocyte globulin (ATG).

### Survival after allogeneic SCT and post-transplant therapy

**Fig. 1:**
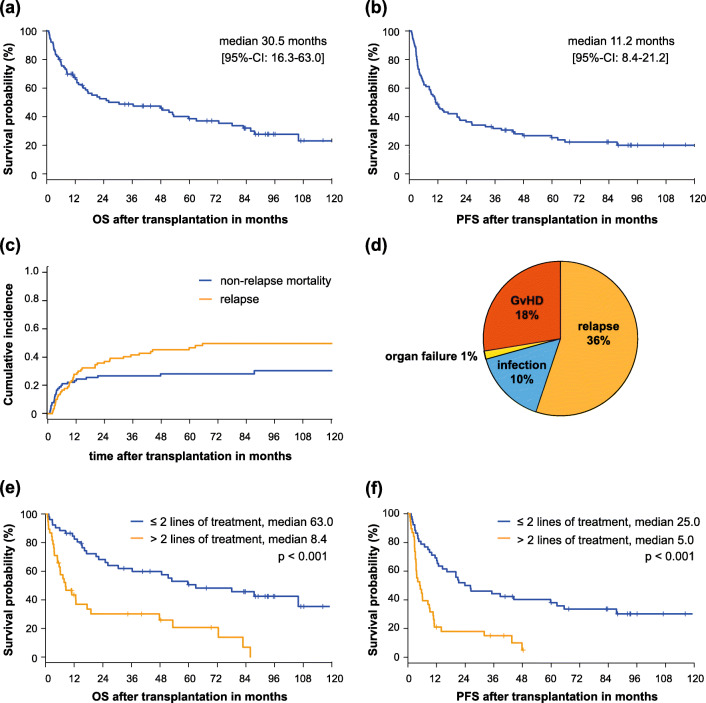
**a** Overall survival (OS) with median and confidence interval (CI). **b** Progression-free survival (PFS) with median and CI. **c** Cumulative incidence of relapse and non-relapse mortality. **d** Causes of death following allogeneic stem cell transplantation. **e**, **f** OS and PFS for subgroups stratified on the number of therapy lines prior to transplantation (≤ 2 versus > 2)

With a median follow-up of 76 months, we observed a median OS and PFS of 30.5 (95% CI 16.3–63.0) and 11.2 (95% CI 8.4–21.2) months, respectively (Fig. 1a, b). In the group receiving allogeneic SCT in first line (*n* = 26), median OS and PFS were significantly longer with 87.5 (95% CI 48.7–not reached (n.r.)) and 36.9 months (95% CI 20.0–n.r.), respectively.

Forty-three patients (48%) experienced relapse or progression. The cumulative incidence of relapse at 1, 3, and 5 years after allogeneic SCT was 27.8% (95% CI 19.9–38.8), 41.5% (95% CI 32.4–53.1), and 46.7% (95% CI 37.2–58.4), respectively (Fig. 1c). There was a small further increase of cumulative incidence of relapse from the third year onwards, with 50% at 10 years. Median survival after first relapse/progression was 15.6 months (95% CI 8.5–28.9). The cumulative incidence of NRM at 1, 3, and 5 years was 23.3% (95% CI 16.0–33.9), 26.7% (95% CI 19.0–37.7), and 28.1% (95% CI 20.1–39.2), respectively (Fig. 1c).

Causes of death, as analyzed according to a validated scheme by Copelan et al. [20], are depicted in Fig. 1d. Acute GvHD grades II–IV occurred in 44 patients (49%), and moderate-to-severe chronic GvHD (cGvHD) was documented in 31 patients (34%).

Eleven patients received low-dose lenalidomide as maintenance therapy after allogeneic SCT. Twelve patients received a median of four donor lymphocyte infusions (DLI) (range 1–8) after a median of 19 months (range 6–58) for serological relapse. In five of those patients, long-term remission of more than 3 years from the date of first DLI was documented. Other post-transplant relapse treatment strategies included lenalidomide, bortezomib, thalidomide, pomalidomide, daratumumab, carfilzomib, panobinostat, and chemotherapy-based regimens. In the majority (70%), at least partial remission following salvage therapy could be achieved.

### Subgroup analysis

To investigate characteristics of those patients benefiting most from allogeneic SCT, we performed a subgroup analysis. Stratified on the number of previous therapies, the group receiving not more than two lines of therapy prior to allogeneic SCT (*n* = 51) had a median OS and PFS of 63.0 (95% CI 30.5–n.r.) and 25.0 (95% CI 14.5–65.6) months, respectively, versus 8.4 (95% CI 5.7–47.8) and 5.0 (95% CI 3.4–10.5) months in the group receiving more than two lines of therapy (*p* < 0.001; Fig. 1e, f). Interestingly, the cumulative incidence of NRM at 12 months was significantly different with 13.5% (95% CI 6.8–26.8) in the first vs. 36.8% (95% CI 24.3–55.9) in the latter group, respectively (*p* = 0.02). We observed inferior OS and PFS in patients with an HLA-nonidentical donor. Stratification on high-risk cytogenetics, age, type of donor (MRD vs. matched unrelated donor (MUD)), conditioning scheme (MAC vs. reduced intensity conditioning (RIC)), use of ATG, or disease control before allogeneic SCT did not show any statistically significant differences.

Important risk factors and factors with significance in univariate analysis were included into the multivariate Cox model (Table 2). Again, receiving more than two lines of therapy prior to transplantation was significantly associated with lower OS and PFS. This association was still present in the subgroup of patients who received allogeneic SCT in r/r disease. In this subgroup, PFS was significantly reduced in patients receiving RIC. In multivariate analysis, survival was not associated with age or disease activity at the time of allogeneic SCT.

**Table 2:** Multivariate Cox regression analysis of the full cohort versus the subgroup with allogeneic stem cell transplantation in relapsed/refractory (r/r) disease

	OS - full cohort		OS - r/r disease		PFS - full cohort		PFS - r/r disease	
Prognostic factor	HR [95% CI]	*p* value	HR [95% CI]	*p* value	HR [95% CI]	*p* value	HR [95% CI]	*p* value
Conditioning RIC vs. MAC	1.06 [0.79–1.41]	0.7	1.37 [0.99–1.89]	0.06	1.10 [0.84–1.45]	0.5	1.42 [1.03–1.94]	0.03
Age at transplantation ≥ 51 vs. < 51 years	0.72 [0.42–1.23]	0.2	0.62 [0.34–1.14]	0.1	0.84 [0.52–1.37]	0.5	0.75 [0.42–1.32]	0.3
Lines of therapy > 2 vs. ≤ 2	3.68 [2.02–6.70]	< 0.001	2.72 [1.39–5.31]	0.003	3.69 [2.09–6.50]	< 0.001	3.15 [1.64–6.05]	< 0.001
Disease activity PR/VGPR/CR vs. SD/PD	0.77 [0.35–1.66]	0.5	1.20 [0.49–2.93]	0.7	0.63 [0.33–1.23]	0.2	0.72 [0.33–1.53]	0.4

Hazard ratios (HRs) greater or less than 1.0 indicate an increased or decreased risk, respectively, of an event for the first category listed. *HR*, hazard ratio; *CI*, confidence interval; *PR*, partial remission; *VGPR*, very good partial remission; *CR*, complete remission; *SD*, stable disease; *PD*, progressive disease

### Immunoglobulin reconstitution after transplantation

**Fig. 2:**
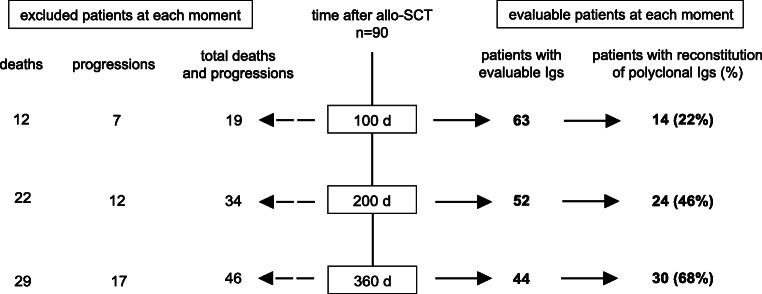
Kinetics of polyclonal immunoglobulin (Ig) reconstitution

**Fig. 3:**
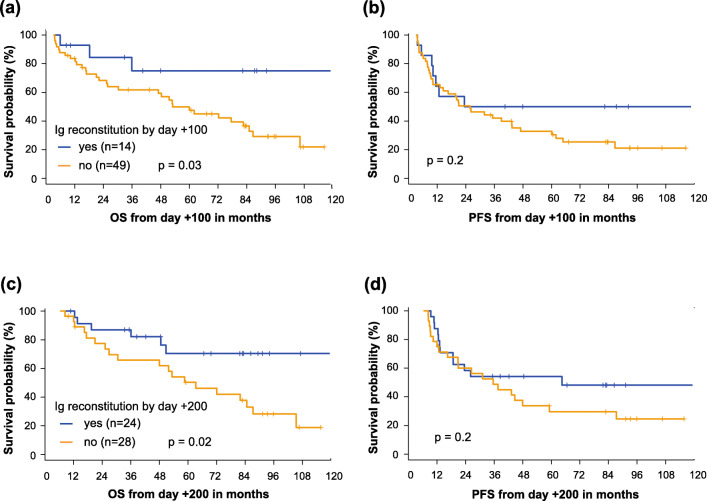
**a**, **b** Conditional overall survival (OS) and progression-free survival (PFS) from the landmark day + 100 following allogeneic stem cell transplantation (SCT) in non-relapsed patients, stratified for presence of immunoglobulin (Ig) reconstitution by day + 100. **c**, **d** Conditional OS and PFS from the landmark day + 200 following allogeneic SCT in non-relapsed patients, stratified for presence of Ig reconstitution by day + 200

We investigated the association of immunoparesis with survival following allogeneic SCT. At 100, 200, and 365 days, 63, 52, and 44 patients without relapse had evaluable Ig measurement, respectively (Fig. 2). By univariate landmark analysis, immunoparesis was associated with inferior OS at 100 days (median n.r. vs. 49.6 months, respectively) and 200 days (median n.r. vs. 56.7 months, respectively) but not at 365 days (Fig. 3a, c). Immunoparesis was not associated with PFS (Fig. 3b, d). Significant differences in cumulative incidences of relapse or NRM could not be observed in patients with or without immunoparesis (Table 3). Of note, patients with immunoparesis at 200 days were more likely to receive immunosuppressive therapy. Therefore, immunosuppressive therapy was considered as important confounder variable affecting the association between immunoparesis and OS. Including this information into the multivariate analysis, inferior OS was observed in patients with immunoparesis at 200 days (HR 3.22, 95% CI 1.14–9.11), whereas use of total body irradiation (TBI) or immunosuppressive therapy was not associated with survival (Table 4).

**Table 3:** Characteristics and outcome of patients alive and progression-free at 200 days after allogeneic stem cell transplantation

		Immunoparesis at 200 days after SCT (*n* = 28)	Ig reconstitution at 200 days after SCT (*n* = 24)	*p* value for difference
Age at SCT in years, median (range)		50.2 (35–60)	52.9 (39–68)	*p* = 0.05
TBI conditioning		11	4	*p* = 0.07
Lenalidomide maintenance therapy		4	6	*p* = 0.3
Immunosuppressive therapy at 200 days after SCT (%)*		18 (35)	5 (10)	*p* = 0.002
No immunosuppressive therapy at 200 days after SCT (%)*		10 (20)	18 (35)	
OS at 2 years after SCT (95% CI)		77,4 (63.0–95.1)	87.0 (74.2–100)	
OS at 5 years after SCT (95% CI)		50.3 (34.3–73.7)	70.4 (52.7–94.0)	
Median OS from 200 days after SCT (95% CI)		56.7 (24.0–n.r.)	n.r. (n.r.–n.r.)	*p* = 0.02
PFS at 2 years after SCT (95% CI)		60.0 (44.2–81.5)	58.3 (41.6–81.8)	
PFS at 5 years after SCT (95% CI)		29.5 (16.4–53.3)	54.2 (37.5–78.3)	
Median PFS from 200 days after SCT (95% CI)		29.0 (14.3–80.7)	57.9 (12.2–n.r.)	*p* = 0.2
Cumulative incidences (95% CI)				
RR	1 year after SCT	17.9 (8.1–39.5)	12.5 (4.3–36.0)	*p* = 0.9
	3 years after SCT	36.6 (22.3–60.0)	41.7 (26.0–66.9)	
	5 years after SCT	52.1 (36.1–75.0)	41.7 (26.0–66.9)	
NRM	1 year after SCT	3.6 (0.5–24.5)	0 (0)	*p* = 0.1
	3 years after SCT	14.6 (5.9–36.2)	4.2 (0.6–28.4)	
	5 years after SCT	18.4 (8.3–40.6)	4.2 (0.6–28.4)	
Cause of death (*n*)				
Relapse		13	5	
Infection		1	1	
GvHD		5	0	

*SCT*, allogeneic stem cell transplantation; *Ig*, immunoglobulin; *TBI*, total body irradiation; *CI*, confidence interval; *RR*, relapse rate; *NRM*, non-relapse mortality; *GvHD*, graft-versus-host disease

*Data available for 51/52 patients

**Table 4:** Multivariate Cox regression analysis for OS for patients alive and progression free at 200 days after allogeneic stem cell transplantation (*n* = 51)

Variable	Frequency	HR	95% CI	*p* value
Immunosuppressive therapy	23 vs. 28	0.98	0.42–2.29	0.96
Immunoparesis	28 vs. 23	3.22	1.14–9.11	0.03
TBI conditioning	15 vs. 36	1.44	0.63–3.28	0.39

*HR*, hazard ratio; *CI*, confidence interval; *TBI*, total body irradiation

As the absolute lymphocyte count has been shown to be associated with survival after allogeneic SCT, we aimed to rule out that immunoglobulin levels were a surrogate for decreased lymphocyte count [13]. In our analysis, total lymphocyte counts did not predict OS or PFS. The prevalence of lenalidomide maintenance therapy was not significantly different in the subgroups with and without immunoparesis at 200 days (*p* = 0.3).

## Discussion

After successful implementation of novel therapeutic agents in MM therapy, the role of allogeneic transplantation needs to be re-determined. The general impact of and in particular the optimal time-point for allogeneic SCT in the sequence of therapies are controversial due to conflicting results of randomized trials, as outlined in a systematic review by Yin et al. [21]. For a subset of younger patients with genetically defined high-risk MM such as 17p deletion, remissions are of limited duration despite continuous therapy with novel agents. For this group of patients, results from randomized trials and retrospective studies are consistent, demonstrating a benefit for allogeneic transplantation [4, 22, 23]. Our retrospective results confirm this evidence, although the subgroup with high-risk cytogenetic aberrations was small (26 patients). This observation underlines the need for transplantation trials stratifying on known risk factors. Moreover, as humanized CAR T cells have been introduced for r/r MM, prospective trials evaluating CAR T cell therapy versus allogeneic transplantation or both maneuvers in sequence might provide new insights [24–26].

The intensity and duration of preceding therapy are associated with outcomes after allogeneic SCT, similar to findings from previous retrospective studies [3, 27]. Patients transplanted in first or second line had superior OS and PFS as compared with patients with 3 and more lines of therapy before transplantation. Recent data could demonstrate how varying selective pressure of therapies gives rise to clonal evolution and chemoresistance [28]. This could be one explanation for the failure of graft-versus-myeloma effect and early relapse in intensively pre-treated patients. In our cohort, the overall relapse rate was slightly lower than in previous reports, exceeding 47% at 5 years. Comparable with existing data, we found only a small further increase of cumulative incidence of relapse from the third year on, indicating that relapse-free long-term survival might be possible [3, 29]. With 23% in the first year, NRM was in the upper range compared with previous reports [30–32], with main causes of non-relapse death being GvHD and infectious complications (Fig. 1d). This finding might be partially due to the relatively high proportion (60%) of MAC regimens included in our analysis, mostly consisting of busulfan- and cyclophosphamide-containing regimens. Notably, NRM was only 13.5% at 12 months in patients transplanted after first- or second-line treatment, possibly mirroring a lower amount of overall toxicity induced by preceding treatment and hence a better capacity to cope with increased toxicity as a result of MAC regimes.

Evidence on post-transplantation consolidation and pre-emptive strategies to reduce the relapse rate is limited to small series. In our study, DLI was successfully applied as an early therapeutic strategy in 12 patients with serological progressive disease and resulted in long-term survival in five cases, confirming previous results showing that DLI is a viable approach [8]. Additionally, 11 patients received lenalidomide maintenance therapy. After relapse, 26 patients received post-transplant salvage therapy, consisting of PIs, IMiDs, and/or new myeloma-directed antibodies. Although most patients were bortezomib- and/or PI-experienced before transplantation, salvage treatment led to remissions in 70%, suggesting that the immunomodulatory effect of therapy might occur irrespective of pre-treatment. Notably, in four patients receiving the more recently approved drugs carfilzomib, pomalidomide, and daratumumab, administration was safe and well-tolerated and led to serological responses in all four cases. To our knowledge, data is scarce on administration of these drugs in the post-allogeneic transplant setting, hence warranting clinical trials.

Recent studies highlighted the prognostic implications of immunoparesis 1 year following autologous transplantation for OS and PFS in MM [14, 16]. Inspired by these findings, we retrospectively assessed the reconstitution of uninvolved polyclonal Ig and the association with survival after allogeneic SCT in patients in remission. We were able to demonstrate the gradual reconstitution of polyclonal Ig after allogeneic SCT in the majority of relapse-free patients (68%) by 1 year after transplantation (Fig. 2). In our institution, supplementation of polyclonal IgG (IVIG) was only occasionally performed in patients with hypogammaglobulinemia and severe or recurrent infections; therefore, reported Ig levels are not likely to be increased artificially. Moreover, as most patients had IgG subtype myeloma, IgA was the predominant uninvolved Ig defining immunoparesis.

Results from our landmark analysis at 200 days after allogeneic SCT suggest immunoparesis as a reflection of delayed B-cellular immune reconstitution being an independent adverse prognostic factor for OS, but not for PFS (Fig. 3; Table 4). Delayed immune reconstitution, GvHD, and prolonged immunosuppressive therapy are potential explanations for immunoparesis, increasing the risk of non-relapse mortality and impeding the graft-versus-myeloma effect. In our analysis, the worse outcome for patients with immunoparesis at 200 days might be partly explained by the prolonged use of immunosuppressive therapy in this subgroup and, consequently, a higher risk of NRM due to infection or GvHD (Table 3). However, differences in NRM rates between patients with and without immunoparesis could not be observed.

An additional underlying mechanism for our observation might be sub-clinical relapse with expansion of bone marrow myeloma cells and subsequent suppression of normal plasma cells via bone marrow microenvironment factors that are not yet comprehensively understood [33, 34]. Consistent with our findings, Schmitz et al. have observed the emergence of secondary monoclonal gammopathy of undetermined significance post-transplantation as a reflection of strong humoral immune response being an independent predictive factor for PFS and OS [35]. On the other hand, deficiency of naive and transitional B cells in an early phase after transplantation is postulated to facilitate alloreactivity and development of cGvHD [36]. There are different immunologic presentations of cGvHD, with elevated, decreased, or normal Ig values in distinct subgroups, mirroring a complex distortion of B cell homeostasis [37–40]. In a study of Ayuk et al., elevated levels of IgG were associated with adverse OS in patients with cGvHD; however, myeloma patients were excluded from the analysis [41]. We propose routine monitoring and prospective studies of polyclonal immunoglobulins and cellular immunity in MM patients after transplantation in order to gain insights into immune reconstitution. The prognostic consequences of immunoparesis remain to be further elucidated.

Limitations of our study are the retrospective single-center design, the variability of conditioning and post-transplant therapeutic regimens, and small subgroups; therefore, results from our subgroup analysis should be interpreted with caution.

We confirm previous reports regarding the dismal outcome of delayed transplantation in refractory disease. For carefully selected high-risk patients, allogeneic stem cell transplantation in earlier phases of disease, e.g., after re-induction following first relapse, might offer benefits regarding long-term survival. Monitoring of polyclonal immunoglobulins in the first year after transplantation could potentially identify patients at risk for death without progression or relapse who need closer follow-up.

## Data Availability

All data were retrieved by the institutional patient records.
